# Exploring the feasibility and acceptability of a mixed-methods pilot randomized controlled trial testing a 12-week physical activity intervention with adolescent and young adult cancer survivors

**DOI:** 10.1186/s40814-019-0530-6

**Published:** 2019-12-20

**Authors:** Amanda Wurz, Jennifer Brunet

**Affiliations:** 1 School of Human Kinetics, University of Ottawa, 125 University Private, Montpetit Hall, Room 339, Ottawa, Ontario K1N 6 N5 Canada; 2 Present address: Faculty of Kinesiology, University of Calgary, Calgary, Alberta Canada; 3 Cancer Therapeutics Program, Ottawa Hospital Research Institute, The Ottawa Hospital, Ottawa, Ontario Canada; 4 Institut du savoir Montfort, Hôpital Montfort, Ottawa, Ontario Canada

**Keywords:** Adolescent and young adult, Oncology, Exercise, Feasibility, Acceptability, Pragmatic approach

## Abstract

**Background:**

Adolescent and young adult (AYA) cancer survivors (i.e. individuals diagnosed with cancer between 15 and 39 years and who completed treatment) may benefit from physical activity. Yet, few researchers have explored the effects of physical activity on physical and psychological outcomes among AYA cancer survivors. A pilot study exploring the feasibility and acceptability of a physical activity intervention and proposed trial methods to inform a definitive randomized controlled trial (RCT) is therefore necessary to fill this gap.

**Methods:**

A two-arm, mixed-methods pilot RCT was conducted. Participants were randomized to a wait-list control group or a 12-week physical activity intervention comprised of 4 weekly aerobic and strength training sessions (intervention group). Feasibility measures included: number of AYA cancer survivors referred/self-referred, eligible, and recruited, retention to the trial (i.e. assessment completion), adherence to the physical activity intervention, and percentage of missing data for baseline (week 0), mid- (week 6), and post-intervention assessments (week 12). The acceptability of trial methods (all participants) and the intervention (intervention group only) was assessed via qualitative interviews post-intervention.

**Results:**

Over a 12-month period, 31 AYA cancer survivors were referred/self-referred and 16 were eligible and consented to participate. Retention to the trial was 94% and adherence to the physical activity intervention ranged from 50 to 92%. With the exception of the assessment of aerobic capacity and directly measured physical activity behaviour, there were no missing data. Participants generally reported being satisfied with the trial methods and intervention; however, issues related to delivery of the physical activity intervention were identified.

**Conclusions:**

The methods and intervention piloted require modification and further pilot testing in advance of a definitive RCT. Recruitment strategies identifying a greater number of younger AYA cancer survivors who have different types of cancers and who lack motivation to participate in physical activity-based studies should be explored. Refining the assessments of directly measured physical activity behaviour and aerobic capacity and incorporating behavioural support into the intervention may improve feasibility and acceptability. This study highlights the value of doing pilot work and provides critically useful data that can be used to refine studies seeking to assess causation and optimize physical activity interventions for AYA cancer survivors.

**Trial registration:**

clinicaltrials.gov, NCT03016728. Registered January 11, 2017.

## Background

Adolescent and young adult (AYA) cancer survivors (i.e. individuals diagnosed with cancer between the ages of 15 and 39 years who have completed treatment [1]) face a range of negative physical (e.g. body composition changes, disfigurement, tissue damage, morbidity, premature mortality) and psychological effects (e.g. reduced self-esteem, lowered quality of life, anxiety [2–4]). Though these adverse effects are reported regardless of age at diagnosis, researchers have found that AYA cancer survivors experience a greater symptom burden than their older counterparts diagnosed with similar cancers [5–7]. This is due, in part, to the transitional period AYA cancer survivors are in when diagnosed that necessitates managing cancer and its effects while navigating critical developmental milestones (e.g. moving from childhood to adulthood physically, psychologically, socially, financially, and educationally [8]). Given AYA cancer survivors’ age at diagnosis and the subsequent number of life-years affected by cancer-related sequelae, minimizing the negative impact for this population while promoting longevity has been identified as a priority [9, 10]. Despite this, few interventions that have the potential to promote length and quality of life have been developed, implemented, and evaluated with AYA cancer survivors.

### Physical activity for AYA cancer survivors

There is considerable evidence from experimental studies showing that participation in physical activity yields numerous physical and psychological health benefits for adult cancer survivors [11–13]. Commonly reported benefits include improved muscular strength and endurance, aerobic capacity, physical functioning, mood, self-esteem, and quality of life [11–13]. As such, many researchers have begun to explore the role of physical activity for AYA cancer survivors. Early evidence suggests physical activity is associated with a range of physical and psychological benefits, similar to those reported among older adult cancer survivors [14, 15]. Notwithstanding the contributions from these studies, the collective body of research has limitations. Specifically, researchers have typically assessed a narrow range of outcomes with homogenous samples and have primarily used cross-sectional study designs grounded in the positivist paradigm [16]. There is a need for research that incorporates a range of physical and psychological outcomes, adopts longitudinal or intervention study designs, and utilizes different paradigms (e.g. interpretivist [17–19]). As well, potential mediators and moderators of the relationship between physical activity and physical and psychological outcomes remain under explored [17–19], which prevents an understanding of how physical activity might be beneficial and under what circumstances desired outcomes may be maximized for this population.

### Mediators and moderators of the relationship between physical activity and physical and psychological outcomes

Identifying the mechanisms through which physical activity exerts its benefits can provide researchers with meaningful targets to optimize intervention effects. The exercise and self-esteem model (EXSEM [20]) has been used to achieve these aims and test how physical activity impacts physical and psychological outcomes in samples drawn from the general population [21, 22]. Within the EXSEM, Sonstroem and Morgan [20] suggest that participating in physical activity leads to changes in one’s physical fitness (i.e. physical measures such as weight status, muscular strength and endurance, aerobic capacity, etc.) and self-efficacy (i.e. confidence regarding one’s ability to successfully engage in physical activity). Improved physical fitness and self-efficacy then lead to improved physical self-perceptions (i.e. self-evaluation of one’s overall physical condition and fitness), which enhances physical self-esteem (i.e. subjective evaluation of the function and appearance of one’s body) and subsequently global self-esteem (i.e. subjective evaluation of one’s self-worth). Among older breast cancer survivors, physical fitness, self-efficacy, and physical self-perceptions have been shown to mediate the relationships between physical activity and physical and global self-esteem [23–25]. Based on research showing that AYA cancer survivors who are more active report greater self-esteem [26, 27] and that participating in physical activity may positively impact one’s self-efficacy [28] and appearance satisfaction [29], there is reason to believe that these findings may extend to AYA cancer survivors. Moderation hypotheses can also be drawn based on a cross-sectional study that found that self-efficacy for physical activity interacted with physical self-perceptions to promote psychological outcomes among AYA cancer survivors [30]. However, the mediators and moderators embedded within the EXSEM remain untested with AYA cancer survivors in trials using experimental designs. Studies testing outcomes of physical activity interventions among AYA cancer survivors and exploring mediators and moderators could generate useful information to guide physical activity recommendations and identify targets for future physical activity interventions seeking to promote physical and psychological outcomes in this cohort.

### The continuum of evidence

Though a definitive randomized controlled trial (RCT) is warranted to address questions of causation and elucidate mechanisms, it is not indicated at this time [31]. This is because there is a lack of research exploring the effects of physical activity interventions delivered in-person (i.e. face-to-face) to AYA cancer survivors. As a result, markers of feasibility (e.g. recruitment, retention, adherence) and acceptability (e.g. satisfaction with trial methods and intervention components) remain unknown. Moreover, there is little information regarding recruitment, retention, and adherence metrics for physical activity research in this population. Collecting this information is vital to conserve valuable research resources and enhance the likelihood of successful definitive RCTs [31, 32]. Following the continuum of evidence put forth by Campbell et al. [31], a pilot RCT is the necessary next step towards examining if and how physical activity improves physical and psychological outcomes among AYA cancer survivors.

### Current study

A two-arm, mixed-methods RCT was developed to test the effects of a 12-week physical activity intervention. To lay the foundation for a future definitive RCT, a pilot RCT assessing the feasibility (defined as recruitment over a 12-month period, retention, adherence, and completeness of data) and acceptability (defined as satisfaction) of trial methods (e.g. randomization, procedures) and the intervention (e.g. intervention delivery) was required.

## Methods

### Study design

This study was a two-arm, mixed-methods pilot RCT designed to test a 12-week physical activity intervention on a range of physical and psychological outcomes among AYA cancer survivors. The protocol was registered in the ClinicalTrials.gov database (NCT03016728), and was approved by the Ottawa Health Science Network, Children’s Hospital of Eastern Ontario, University of Ottawa, and Royal Ottawa Mental Health Centre Research Ethics Boards. The reporting standards for pilot trials put forward by Consolidated Standards of Reporting Trials (CONSORT [33]) were followed in the preparation of this manuscript (see Additional file 1).

### Sample

The target sample consisted of AYA cancer survivors who: (1) were diagnosed with cancer between the ages of 15 and 39 years; (2) had completed cancer treatment within the past 5 years; (3) showed no evidence of progressive or recurrent disease or of secondary or second cancers; (4) were inactive or insufficiently active (assessed using a single-item screening question: ‘Are you currently engaging in moderate physical activity, that is activity that increases your heart rate and causes you to sweat, > 3 days/week?’); (5) were medically cleared to participate in physical activity^1^; and (6) were able to read, understand, and provide informed consent in English. AYA cancer survivors were not eligible if they: (1) had physical impairments precluding participation in physical activity and/or (2) were unwilling or unable to provide informed consent.

### Procedures

AYA cancer survivors were recruited across a 12-month period starting in September 2017 through healthcare provider referral (wherein eligible AYA cancer survivors were first screened and then approached by their healthcare provider to obtain consent for the first author to contact) and snowball sampling (wherein potentially eligible AYA cancer survivors self-screened and then contacted the first author). A 12-month period was specified a priori so as to capture seasonal variation that may affect trial and intervention feasibility and/or acceptability, and thus better inform the timeline for a definitive RCT. Following confirmation of eligibility and obtaining informed consent, participants completed a baseline assessment (week 0) comprised of physical tests, a survey, a qualitative interview, and wearing an accelerometer for 7 consecutive days. Afterwards, participants were randomly assigned to either the intervention or wait-list control group by an independent researcher who used a web-based random number generator. Participants completed mid- (week 6) and post-intervention assessments (week 12), which resembled the baseline assessment. All assessments were conducted by the first author at a private location of participants’ choosing. At study cessation participants were entered into a draw to win a $250 gift card, regardless of retention to the trial and/or adherence to the intervention.

### Physical activity intervention

The physical activity intervention was developed across a 6-month period. Intervention components were first selected based on recent systematic reviews [11–13, 34, 35], clinical guidelines [36], physical activity recommendations [37], behaviour change literature [38–40], and population-specific preferences for physical activity [41–44]. This was augmented by eliciting opinions from an advisory board comprised of three AYA cancer survivors (who met the eligibility criteria outlined above), three allied healthcare providers (*n* = 1 Kinesiologist; *n* = 2 Certified Exercise Physiologists), and two oncologists. The result was a 12-week physical activity intervention using a pragmatic approach, wherein flexibility was prioritized to minimize participant burden.

Participants assigned to the intervention group received a 12-week individualized physical activity program and were lent equipment for weeks 1–12 (i.e. hand weights, resistance bands). They were also provided with a fitness bag that had a water bottle, socks, sweat towel, and yoga mat to minimize barriers related to access. Participants could keep the bag and its contents post-intervention. The program consisted of 4 weekly sessions lasting for 25–45 min/session (see Table 1 for an overview). Two of these sessions focused on strength activities and were supervised by the first author^2^ for weeks 1–6 (e.g. squats, lunges, shoulder press, bicep curls). During these sessions, participants were taught proper form and technique and were provided rationale for each intervention component to enhance their knowledge. They were also offered various modifications and supported in choosing intensities that were right for them to enhance their feelings of confidence and competence. Based on participants’ preferences, sessions took place at participants’ home (*n* = 40 sessions), a local cancer survivorship centre (*n* = 15 sessions), or the University of Ottawa (*n* = 4 sessions). In weeks 7–12, participants were instructed to continue engaging in strength training two times/week unsupervised. Throughout weeks 1–12, participants were asked to participate in two unsupervised sessions/week focused on aerobic activities (e.g. walking, rowing, indoor/outdoor bicycling, jogging) between 40 and 75% of their estimated heart rate reserve. Participants were provided with a Polar A300 activity monitor with a heart rate strap and taught how to use a 10-point Perceived Exertion Scale as a means of verifying aerobic session prescription and teaching them how to self-monitor. The volume and intensity of each aerobic and strength training session were modifiable depending on how participants felt that day and was progressed over the course of the 12-week intervention on an individual basis so as to ensure participants experienced success with the program while acquiring new skills.

**Table 1:** Overview of the 12-week physical activity program

	Aerobic training						Strength training	
Week	1, 2	3, 4	5, 6	7, 8	9, 10	11, 12	1–6	7–12
Days/week	2						2 (non-consecutive)	
Warm up (min)	5						5	
Training (min)	15	20	25	30	30	30	15–20	15–20
Target intensity	40–60 %HRR	40–60 %HRR	40–60 %HRR	40–60 %HRR	60–75 %HRR	60–75 %HRR	1–2 sets 8–12 RM	2–3 sets 6–10 RM
Type	Any self-selected aerobic physical activity (e.g. walking, rowing, indoor/outdoor bicycling, jogging)						8–10 full body exercises	
Cool down (min)	5						5–10 (comprised of 8–10 full body flexibility exercises)	
Supervised	No						Yes	No

*HRR*, heart rate reserve; *min*, minutes; *RM*, repetition maximum

### Wait-list control group

Participants assigned to the wait-list control group were advised to continue with their usual routine for weeks 1–12. After their post-intervention assessment (week 12), participants received the same 12-week intervention, materials, and equipment as the intervention group.

### Measures

#### Feasibility (throughout the trial)

To assess feasibility, the number of AYA cancer survivors referred, eligible, recruited/not recruited (reasons for non-recruitment), retention to the trial, adherence to the physical activity intervention, and percentage of missing data were collected. Source of referrals were tracked and recruitment rate was defined as the number of eligible participants who enrolled in the trial out of the number of eligible AYA cancer survivors who self-referred or were referred. Retention rate was defined as the number of participants completing all three assessments. Adherence rates were defined as the number of supervised strength sessions engaged in out of 12 (weeks 1–6), unsupervised strength sessions engaged in out of 12 (weeks 1–6), and unsupervised aerobic sessions engaged in out of 24 (weeks 1–12). To collect this information, participants in the intervention group completed weekly physical activity logbooks across weeks 1–12. Completeness of quantitative data and participation in interviews were also examined. Missingness was defined as percentage of missing data on each measure and overall.

As recommended for pilot studies [32, 45], a priori targets for each feasibility outcome were set using relevant literature [46–49] and the authors’ own clinical experience: (1) 36–48 AYAs referred/self-referred over 12 months, (2) > 70% of eligible AYA cancer survivors agree to be enrolled, (3) ≥ 75% of participants complete baseline, mid- and post-intervention assessments, (4) each participant assigned to the intervention group completes > 75% of the prescribed physical activity intervention, and (5) < 10% missing data overall.

#### Acceptability (week 12)

All participants answered questions related to the acceptability of trial methods (e.g. satisfaction with randomization, assessments, procedures) at their post-intervention assessment (week 12). For those in the intervention group, this was commensurate with when they finished the intervention. For those in the wait-list control group, this was commensurate with the end of their 12-week waiting period (before they received the intervention). Additional questions related to intervention acceptability (e.g. satisfaction with intervention components [delivery, modality, length, duration]) were asked to participants in the intervention group only at their post-intervention assessment (week 12).

#### Adverse event monitoring (throughout the trial)

Participants’ were instructed to self-report adverse events to the first author who had a standardized reporting form (e.g. date, severity, timing, site/location, duration, clinical action taken, outcome). None were reported.

#### Personal and medical factors (week 0)

Participants self-reported a range of personal (i.e. age, sex, annual household income, education attainment, school/work status) and medical factors (i.e. cancer diagnosis, treatment protocol, time since treatment, co-morbid conditions), which were used as a means of describing the sample and for tailoring/individualizing participants’ physical activity intervention.

#### Physical activity behaviour (week 0, week 6, week 12)

Self-reported physical activity was assessed using a modified version of the Leisure Time Exercise Questionnaire [50, 51] that has been described elsewhere [30]. Directly measured physical activity was assessed using an accelerometer (Actigraph wGT3XP-BT; Actigraph, LLC, Pensacola, Florida) and was managed with ActiLife v6.13.3 software using established wear time criteria [52] and activity count cut-points [53].

#### Physical outcomes (week 0, week 6, week 12)

Participants’ physical functioning was assessed via a battery of physical tests measuring body composition using a Portable HR-200 height rod and Tanita TBF-310 GS scale using bioelectrical impedance, musculoskeletal strength using the combined grip strength of the right and left hands assessed with a handheld dynamometer [54], muscular endurance using the 30-s sit to stand test [55], resting blood pressure using a blood pressure monitor (HealthSmart Digital Blood Pressure Monitor)^3^, and aerobic capacity using the 6-min walk test (6MWT [54]).

#### Psychological outcomes (week 0, week 6, week 12)

Psychological outcomes were assessed using a self-report survey measuring self-efficacy for physical activity using a modified single-item version of the Exercise Self-Efficacy scale [56] described elsewhere [30]; physical self-perceptions using the Physical Self Description Questionnaire Short Form (PSDQ-S) subscales of strength, endurance, appearance, and body fat (3 items/subscale [57]); physical self-esteem using the PSDQ-S subscale of physical self-esteem (3 items [57]); and global self-esteem using the Rosenberg Global Self-Esteem Scale (10 items [58]). Across all surveys used to assess psychological outcomes, higher scores reflect more positive outcomes.

#### Qualitative interviews (week 0, week 12)

Participants’ perspectives of their physical activity, self-efficacy for physical activity, physical self-perceptions, physical self-esteem, and global self-esteem (i.e. the EXSEM variables) were obtained through semi-structured interviews at baseline (week 0) and post-intervention (week 12). For participants in the intervention group, this was commensurate with the time immediately prior to and after receiving the intervention.

For participants in the wait-list control group, this was commensurate the time immediately prior to and after their waiting period. Interviews were guided by an interview schedule containing a series of open-ended questions and probes to encourage participants to provide more detail or clarify what they were saying. Data pertaining to perceived changes in EXSEM variables are not reported herein, but will be published in forthcoming work as they are outside of the scope of the present study, which was to assess the feasibility and acceptability of the trial methods and intervention.

### Sample size

No formal sample size calculation was performed based on the study objectives.

### Data analysis

We conducted quantitative and qualitative analyses. Descriptive statistics consisted of frequencies, means and standard deviations (*SD*), and 95% confidence intervals for normally distributed data, whereas medians, interquartile ranges, and 95% confidence intervals were computed for non-normally distributed data. All descriptive statistics were estimated using IBM SPSS (Version 25 [59]). These data were used to describe the sample and report on feasibility outcomes. Content analysis of the transcribed acceptability data from the post-intervention (week 12) interview was conducted to ascertain acceptability outcomes [60].

## Results

### Participants

The personal and medical characteristics of participants at baseline are presented in Table 2. At week 0, participants were 32.84 (*SD* = 7.93) years old and had completed treatment for cancer 2.23 (*SD* = 1.15) years prior. On average, participants were diagnosed with cancer at 29.64 (*SD* = 7.73) years of age and most had received a diagnosis of breast cancer (*n* = 7; 44%). Others had received a diagnosis of ovarian cancer (*n* = 2; 13%), rhabdomyosarcoma (*n* = 1; 6%), biphasic peritoneal mesothelioma (*n* = 1; 6%), gastric cancer (*n* = 1; 6%), osteosarcoma (*n* = 1; 6%), soft tissue sarcoma (*n* = 1; 6%), colorectal cancer (*n* = 1; 6%), or Hodgkin’s lymphoma (*n* = 1; 6%). Half of the sample (*n* = 8; 50%) reported managing at least one other physical or psychological health condition (e.g. asthma, neurofibromatosis, blood clots, hypothyroidism, anxiety, depression).

**Table 2:** Characteristics of study participants at baseline

Group	Sex	Current age (years)	Age at diagnosis (years)	Type of cancer diagnosed	Time since treatment completion (years)	Annual household income^a^	Education attainment	School/work status
PA	F	37	35	Ovarian	1.80	> 100,000	Completed graduate school	Full-time employment
WLC	F	32	30	Breast	1.04	Prefer not to answer	Some university/college	Disability
PA	M	22	17	Rhabdomyosarcoma	3.94	> 100,000	Completed university/college	Full-time employment
PA	F	34	31	Biphasic peritoneal mesothelioma	2.98	20–39,999	Some university/college	Disability
WLC	F	39	34	Breast	4.43	> 100,000	Completed university/college	Full-time employment
WLC	F	39	37	Breast	2.23	Prefer not to answer	Completed university/college	Full-time employment
PA	F	30	27	Gastric	2.23	> 100,000	Completed university/college	Part-time employment
WLC	M	22	17	Osteosarcoma	NR	Do not know	Some university/college	Student
WLC	F	35	34	Soft tissue sarcoma	0.76	60–79,999	Completed graduate school	Full-time employment
PA	F	36	34	Colorectal	1.54	20–39,999	Completed university/college	Full-time employment
PA	F	38	34	Breast	1.91	> 100,000	Completed graduate school	Student
WLC	F	39	35	Breast	3.12	> 100,000	Completed university/college	Part-time employment
WLC	F	22	19	Hodgkin's lymphoma	2.03	< 20,000	Some graduate school	Student
PA	F	36	31	Breast	1.70	> 100,000	Some high school	Full-time employment
WLC	F	41	37	Breast	3.37	> 100,000	Some university/college	Full-time employment
WLC	F	15	15	Ovarian	0.38	Do not know	Some high school	Student; part-time employment

^a^Reported as Canadian dollars; *F*, female; *PA*, physical activity intervention group; *M*, male; *NR*, not reported; *WLC*, wait-list control group

Participants’ baseline scores for physical activity behaviour and physical and psychological outcomes are presented in Table 3. Participants self-reported engaging in a median of 60.00 (interquartile range = 93.75) minutes of moderate-to-vigorous intensity physical activity (MVPA)/week, whereas the directly measured physical activity captured that participants were engaging in a median of 44.29 (interquartile range = 57.17) minutes of MVPA/week. On average, participants were classified as overweight (*M*_BMI_ = 29.93 kg/m^2^, *SD* = 9.26). Participants’ scores on the 30-s sit to stand test were below average as compared to normative values for adults > 60 years [61] and most grip strength scores were rated as ‘poor’ (*n* = 7; 44%), followed by ‘good’ (*n* = 3; 19%), ‘fair’ (*n* = 3; 19%), ‘very good’ (*n* = 2; 6%), and ‘excellent’ (*n* = 1; 6%) according to established cut-offs [54]. Blood pressure was considered normal (i.e. systolic less than 120 mmHg and diastolic less than 80 mmHg) for most participants (*n* = 10; 63%). Aerobic capacity scores (i.e. 6MWT) were not computed due to differences in the lengths of the walking track across participants, which can artificially increase/decrease participants’ scores [62]. Finally, participants’ scores on psychological outcomes were ‘moderate’ relative to scale ranges.

**Table 3:** Physical activity and outcome scores for participants at baseline

Variable	Scale range	Intervention (*n* = 7) Mean (*SD*) [95% CI]	Control (*n* = 9) Mean (*SD*) [95% CI]	Total (*n* = 16) Mean (*SD*) [95% CI]
Physical activity behaviour^a^				
Self-reported				
MVPA (min/week)	0–∞	60.00 (140.00)^b^ [0–239.63]	47.50 (41.31) [15.75–79.25]	60.00 (93.75)^b^ [16.72–126.09]
Directly measured				
MVPA (min/week)	0–∞	63.00 (258.22)^b, *n*=5^ [0–370.38]	37.25 (24.34)^, *n*=6^ [11.70–62.79]	44.29 (57.17)^b, *n*=11^ [0–171.66]
Physical outcomes				
BMI (kg/m^2^)^c^	0–∞	31.86 (8.16) [24.31–39.41]	28.43 (10.25) [20.56–36.31]	29.93 (9.26) [25.00–34.86]
Grip strength (kg)	0–∞	56.71 (15.83) [42.07–71.35]	51.00 (9.67) [43.57–58.43]	53.50 (12.60) [46.79–60.21]
Sit to stand (repetitions)	0–∞	12.00 (2.16) [10.00–14.00]	11.22 (2.11) [9.60–12.84]	11.56 (2.10) [10.45–12.68]
Psychological outcomes				
Self-efficacy for MVPA	0–100	85.71 (13.97) [72.79–98.64]	68.89 (24.21) [50.28–87.50]	76.25 (21.56) [64.76–87.74]
Physical self-perceptions				
Strength	1–6	3.95 (0.85) [3.17–4.74]	3.07 (1.02) [2.29–3.86]	3.46 (1.02) [2.91–4.00]
Endurance	1–6	2.67 (1.02) [1.72–3.61]	2.26 (1.21) [1.33–3.19]	2.44 (1.11) [1.84–3.03]
Body fat	1–6	1.86 (0.94) [0.99–2.73]	3.30 (2.26) [1.56–5.04]	2.67 (1.91) [1.65–3.68]
Appearance	1–6	3.86 (0.50) [3.39–4.32]	3.78 (0.44) [3.44–4.12]	3.81 (0.45) [3.57–4.05]
Physical self-esteem	1–6	3.38 (0.68) [2.75–4.01]	2.70 (1.16) [1.81–3.60]	3.00 (1.01) [2.46–3.54]
Global self-esteem	10–40	29.14 (2.19) [27.11–31.17]	28.11 (5.51) [23.88–32.35]	28.56 (4.29) [26.28–30.85]

^a^Light, moderate, and vigorous intensity physical activity data is available upon request; ^b^median and interquartile range; ^c^percent body fat, fat mass, and fat free mass data is available upon request; *CI*, confidence interval; *min*, minutes; *MVPA*, moderate-to-vigorous intensity physical activity; *SD*, standard deviation

### Quantitative results

#### Feasibility

##### Referrals and recruitment

**Fig. 1:**
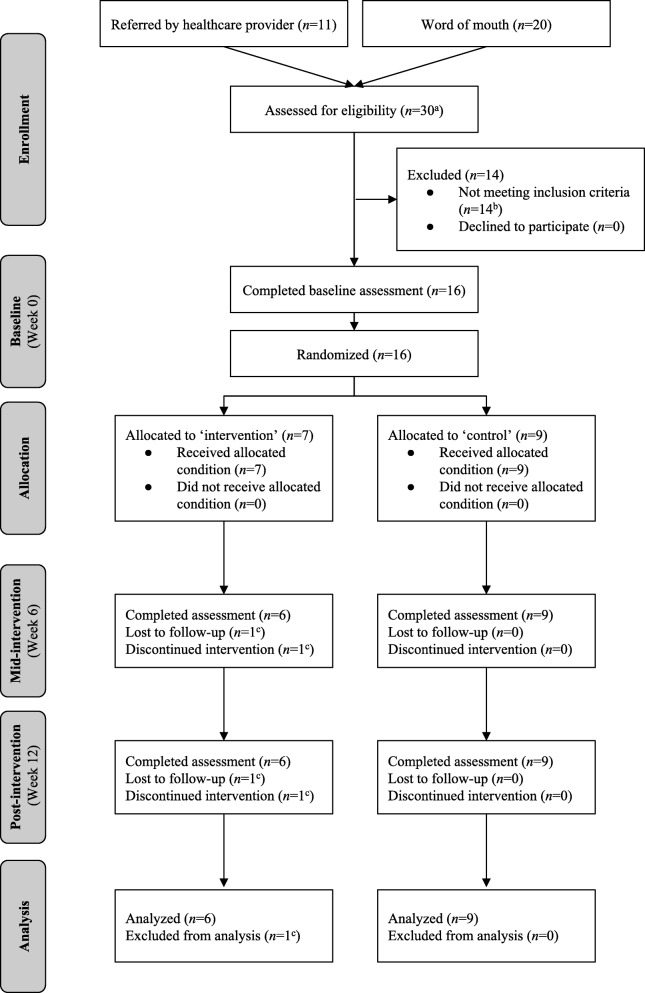
CONSORT flow diagram. ^a^After referral, one individual could not be reached; ^b^reasons for exclusion: meeting physical activity guidelines (*n* = 2), age at diagnosis (*n* = 3), time since treatment (*n* = 5), treatment status (*n* = 4); ^c^withdrew due to family issues that were personally distressing.

In total, 31 AYAs were referred/self-referred across the 12-month period (see Fig. 1). Of these, 30 were assessed for eligibility and 16 were eligible. All who were eligible consented to participate (recruitment rate = 100%). Seven participants were randomly assigned to the intervention group and nine were randomly assigned to the wait-list control group. One participant assigned to the intervention group withdrew from the trial in week 3 and was lost to follow-up (retention rate = 94%).

##### Adherence to physical activity program (intervention group only)

During weeks 1–6, a total of 12 supervised strength sessions were provided. Participants’ adherence to these supervised strength sessions varied from 58 (7/12 sessions) to 92% (11/12 sessions) with an average adherence rate of 82% (mean = 9.83 [*SD* = 1.60]/12 sessions). Five of six participants adhered to ≥ 75%, or ≥ 9 of the 12 sessions. Across all participants, 13 sessions were cancelled due to travel for holidays (*n* = 5), illness (*n* = 6), and work/appointment conflicts (*n* = 2); however, in seven of these instances participants still completed strength sessions on their own unsupervised. During weeks 7–12, participants were instructed to participate in two unsupervised strength sessions/week to total 12 unsupervised strength sessions. Adherence to the unsupervised strength sessions ranged from 50 (6/12 sessions) to 92% (11/12 sessions) with an average adherence rate of 69% (mean = 8.33 [*SD* = 1.97]/12 sessions). Three of six participants adhered to ≥ 75%, or ≥ 9 of the 12 sessions. Most sessions during weeks 7–12 were missed due to being too busy, tired, or ‘lazy’ (*n* = 21); one session was missed due to illness. Throughout weeks 1–12, participants were instructed to participate in two unsupervised aerobic sessions/week to total 24 unsupervised aerobic sessions. Adherence to the unsupervised aerobic sessions ranged from 54 (13/24 sessions) to 88% (21/24 sessions) with an average adherence rate of 76% (mean = 18.17 [*SD* = 2.93]/24 sessions). Four of six participants adhered to ≥ 75%, or ≥ 18 of the 24 sessions. The main reasons unsupervised aerobic sessions were missed were: being too busy or tired (*n* = 23); illness (*n =* 7); work conflicts (*n* = 2); holidays (*n* = 2), and; an unrelated injury (*n* = 1).

##### Missing data

There were no missing data on self-reported physical activity behaviour and psychological outcomes for study completers (*n* = 15). For physical tests, participants completed all measures of body composition, musculoskeletal strength, muscular endurance, and resting blood pressure. However, there were missing data for aerobic capacity and directly measured physical activity behaviour (as assessed using accelerometers). With regard to the former, there were 21 instances of missing aerobic capacity data (out of a possible 45 data points) as six participants could not complete the test at all three time-points due to high blood pressure (a skipping criteria for this assessment was stipulated by the study protocol; *n* = 18) and three participants elected to not complete the test for aerobic capacity at a single time-point due to weather (*n* = 2)^4^ or feeling unwell (*n* = 1). With regard to the latter, there were 13 instances of missing accelerometer data (out of a possible 45 data points) due to: insufficient wear time (i.e. < 3 days of valid wear time; *n* = 8); accelerometer dysfunction (*n* = 1), and; participant error (e.g. wearing the accelerometer incorrectly; *n* = 4). Combined, there were < 10% missing quantitative data across all three time-points for the 15 participants who completed the study. All study completers (*n* = 15) participated in both qualitative interviews.

### Qualitative results

#### Acceptability

During the interviews, no issues surrounding acceptability with the trial methods were raised. Participants said they accepted to be randomized and were highly satisfied with the opportunity to receive a 12-week physical activity program (either immediately or after a waiting period). Participants did not identify any issues related to the assessments or trial procedures. In other words, the number, timing, and duration of assessments and trial-related procedures were deemed acceptable.

Participants randomized to the intervention group held positive regard for the individualized, pragmatic, and progressive nature of the intervention, which was highlighted by [P4] when she said: ‘I liked that we could focus so much on what I needed. Like what I really needed help with was the core stuff because of how damaged it was through my treatment and recovery’, and [P1] when she stated: ‘I thought it was good for adaptation and modification. I also liked the fact it was at our own leisure, and I liked the flexibility of it’. Moreover, participants valued the usefulness of the skills they learned and positively evaluated the performance and skills of the first author who delivered the intervention.

However, participants expressed challenges integrating physical activity into their lives because they had difficulty planning for and overcoming barriers, such as having busy schedules and travel requirements. Further, participants expressed difficulty transitioning from supervised (weeks 1–6) to unsupervised (weeks 7–12) strength training sessions and suggested a more gradual stepped down approach: ‘…instead of going cold turkey at the 6 weeks, if we had gone to one time a week, tapering off to me being on my own. At some point I know I have to be responsible, so I feel it is a 2-way street as well, but if that was built in, it couldn’t hurt’ [P14]. As well, participants commented that more instruction and support for the unsupervised aerobic training (weeks 1–12) would have been helpful: ‘I also felt if we would have had, maybe, I don’t know, like a sheet that would give us ideas of what to do for the aerobic sessions. I know, at least for me, I get bored with just running or walking’ [P7].

## Discussion

The purpose of this two-arm, mixed-methods pilot RCT was to lay the foundation for a future definitive RCT examining if/how and under what circumstances physical activity impacts physical and psychological outcomes in AYA cancer survivors. Though recruitment, retention, and missing data rates were better than targets set a priori and superior to other trials testing distance-based physical activity interventions (e.g. telephone counselling, Facebook [46, 47]), the number of AYA cancer survivors referred/self-referred and participants’ adherence to the intervention were below targets. Findings from this pilot RCT highlight specific aspects of the trial methods and intervention that were feasible as well as those aspects that were not feasible. Modifications to trial methods and intervention components are warranted and require additional piloting before a sufficiently powered definitive RCT can be considered.

The lower than anticipated referrals/self-referrals highlights the difficulty researchers are likely to encounter when seeking to identify and recruit AYA cancer survivors to participate in their physical activity research. Lower referrals/self-referrals may be due, in part, to the rarity of cancer in this age cohort [63, 64] and population-specific barriers to participating in trials (e.g. geographic mobility, small numbers [65]). Moving forward, researchers interested in this line of work may wish to collaborate and conduct multi-site trials to increase sample sizes, ultimately ensuring adequately powered studies. Further, using recruitment strategies beyond those used herein may increase the number of AYA cancer survivors referred or who self-refer and ensure greater diversity (e.g. younger AYA cancer survivors, different types of cancers). For example, researchers could consider mailing/emailing trial brochures using tumour registries, attending hospital rounds and recruiting in-person, and/or attending cancer-related events/groups [66]. Partnering with organizations that typically include AYA cancer survivors in their network and using Internet and social networking are other low-/no-cost options [66].

The higher than anticipated recruitment and retention rates observed are promising for those seeking to deliver physical activity interventions to AYA cancer survivors. Findings re-affirm reports that *some* AYA cancer survivors are eager to participate in lifestyle interventions [43, 67] and want access to health promoting services during this time (i.e. < 5 years post-treatment [68])—widely considered a ‘teachable moment’ in the general cancer literature [69, 70]. However, the rates reported are associated with significant limitations (described in greater detail in the ‘Limitations and considerations’ section) and do little to extend knowledge regarding those AYA cancer survivors who may lack motivation to participate in physical activity-based research.

Missing data are inevitable in trials, yet there were only two measures on which any missing data were documented in this pilot RCT: the objective assessment of aerobic capacity and directly measured physical activity behaviour using accelerometers (see next paragraph for a discussion on this). Nonetheless, this shows the feasibility and acceptability of assessing AYA cancer survivors at multiple times (i.e. baseline, mid-intervention, post-interventions) using a combination of quantitative (directly measured physical activity, physical tests, surveys) and qualitative tools (interview). Moreover, this was found despite the assessments taking > 1.5 h to complete. This may be because the survey and interview included questions previously deemed to be clear, appropriate, and relevant to AYA cancer survivors [71].

In terms of lessons learned with regard to assessment selection, there were missing data on the objective assessment of aerobic capacity. Specifically, the 6MWT (without appropriate monitoring and the supervision of a certified exercise physiologist) was contraindicated due to high blood pressure for six participants during at least one of their assessments, and there were three instances wherein participants chose not to complete an assessment. Further, the distance of the walking track could not be standardized at any time-points across participants due to the location of assessments (e.g. home, apartment hallway). Discrepancies in length can artificially increase/decrease scores on this assessment [62]; thus, 6MWT data for those who could complete the assessment were not reported herein. In the future, researchers and practitioners may wish to omit this assessment or conduct it within a research/healthcare centre to ensure appropriate monitoring, supervision, and standardization. Second, there were missing accelerometer data despite employing recommended strategies to enhance accelerometer compliance (i.e. modelling proper accelerometer use, providing verbal and written instructions, sending reminder messages [72]) and most participants self-reporting in their logbook that they had worn the accelerometer for the required amount of time. This suggests the missing data may not be related to participants’ unwillingness to complete this assessment, but may be related to the process by which missing data points are identified. As there is considerable variability across protocols for determining non-wear (i.e. missing data) and wear time, population-specific protocols are suggested [73]. No protocols have been developed with/for AYA cancer survivors, leaving questions regarding the frequency, pattern, and duration of non-wear and wear times in this population unanswered. Those wishing to include accelerometers in their studies with AYA cancer survivors might consider protocols least affected by wear time and monitor inactivity (e.g. 120 min [74]).

Though participants appreciated the pragmatic nature of the physical activity intervention, adherence was lower than expected, ranging from 50 to 92%. Data from participants’ interviews provided insight into reasons underlying these findings and suggests modifications to the intervention are necessary. This sample had a hard time scheduling and overcoming barriers to engage in physical activity. To help older adult cancer survivors successfully change their behaviour, to achieve desired physical and psychological benefits researchers and practitioners have taught them about the importance of planning physical activity sessions (e.g. scheduling) and have helped them identify barriers to physical activity (e.g. travel) and strategies to overcome them (i.e. problem solving and action planning; e.g. packing a resistance band). Such strategies/skills are known as behaviour change techniques (BCTs [75]). BCTs are the ‘observable, replicable, and irreducible component’ of interventions designed to increase physical activity behaviour [75]. Trials including BCTs have reported greater adherence and behaviour change compared to those that do not [76–78]. Given the critical role BCTs can play in promoting physical activity [79], examining the feasibility and acceptability of integrating BCTs into physical activity interventions for AYA cancer survivors is warranted.

Finally, findings underscore that more pilot work is necessary to optimize the 12-week physical activity program tested. Though the program was individualized based on physical assessment data and designed to maximize change from a physiological perspective, the intervention itself was not individualized according to psychological preferences. Given the varied physical activity preferences of cancer survivors [80] and increasing calls for personalized care in AYA oncology [65], researchers should explore preference-based physical activity trials in this population (e.g. home- or centre-based; yoga or strength training). Similarly, testing different approaches for transitioning from supervised to unsupervised physical activity, wherein survivors receive support that is matched to their needs/wants [81], might be worthwhile. Among older adult cancer survivor, triaged models of physical activity delivery have been shown to be a beneficial and cost-effective means to identify individuals requiring more/less support and offering appropriate care [82–84]. Considering current resource constraints (e.g. personnel, infrastructure) and limited funding available, such approaches may also lower costs needed to translate successful models into care. Moving forward, refining, piloting and testing different study designs (e.g. preference-based trials) and triaged approaches with AYA cancer survivors could answer important research questions, ultimately ensuring this population receives individualized interventions and care. If these and other changes are made, cost description analyses for participants (i.e. time, costs) and researchers (e.g. personnel time and costs, materials/equipment) will be necessary to inform future trials and scalability initiatives.

### Limitations and considerations

Although this pilot RCT provides useful feasibility and acceptability data for researchers wishing to explore physical activity with AYA cancer survivors, there are some major and minor limitations that should be considered. With regard to the major limitations, the recruitment strategies used (i.e. healthcare provider referral and self-referral) did not provide data on the number or characteristics of AYA cancer survivors who were eligible but not interested, and therefore did not consent to be contacted or self-refer. Thus, the recruitment rate presented herein likely reflects only those AYA cancer survivors who intended to participate, and as a result may be higher than if alternative recruitment strategies and/or tracking systems were used. Relatedly, it is possible that the retention and missing data estimates would be lower and higher, respectively, if less motivated AYA cancer survivors were recruited. Despite our efforts to identify inactive or insufficiently active AYA cancer survivors (to limit ceiling effects in a definitive RCT and to explore whether inactive/insufficiently active AYA cancer survivors could be recruited), baseline assessments showed some participants were engaging in physical activity, with one participant meeting current physical activity recommendations. Revisions to this pilot RCTs protocol should incorporate strategies to collect data on eligible but not interested AYA cancer survivors and test targeted strategies to recruit less motivated AYA cancer survivors. At the same time, concerted efforts to recruit a more heterogeneous sample comprised of AYA cancer survivors with different types of cancer, who are younger (i.e. adolescents at time of diagnosis and study), and self-identify as male will be required in advance of a future definitive RCT to ensure generalizability.

With regard to minor limitations, the first author performed the intervention and all assessments (including the acceptability interviews). This may have influenced participants’ responses, such that they responded more positively than they actually wanted to. Moreover, the acceptability interviews were conducted post-intervention (week 12) only; wait-list control group participants were therefore not asked about their experiences with the physical activity intervention since they had not yet started the intervention. Including perspectives of wait-list control group participants might have resulted in additional insights. Fidelity, though tracked by the first author, was not objectively assessed. This has implications and calls to question whether the findings are due to the trial methods/intervention content or other factors. As well, the feasibility and acceptability of including follow-up assessments remains unknown. To better prepare for a fundable definitive RCT and enable examination of physical activity behaviour change maintenance, piloting follow-up assessments will be necessary. Finally, the results presented herein are only applicable to the trial methods and intervention piloted and could be different based on changes to recruitment strategies, assessments (e.g. measurement tools, timing), and the intervention (e.g. frequency, intensity, delivery style, context). Modifications to each may result in additional challenges/barriers to feasibility and acceptability.

## Conclusions

In conclusion, the methods and intervention comprising this two-arm, mixed-methods pilot RCT require modifications and further piloting before being deemed feasible and acceptable. Findings underscore the necessity of conducting pilot RCTs to identify problems in advance of time- and resource-consuming definitive RCTs. Further, results suggest fostering collaborations, working across sites, and using multiple and varied sources of recruitment are necessary to increase the number of AYA cancer survivors referred, approached, and enrolled. Researchers should also ensure study assessments are appropriate and relevant for AYA cancer survivors, and should carefully consider assessments of aerobic capacity and protocols for directly measured physical activity behaviour to reduce missing data. Finally, adding behavioural support through the inclusion of BCTs and testing different models incorporating stepped-down and/or triaged approaches may help some AYA cancer survivors overcome barriers to physical activity and enhance adherence and acceptability. Should these, and other changes, be made, cost description analysis are warranted. This study provides critically useful data that can be used to inform future pilot trials seeking to establish feasibility and acceptability, with the ultimate goal of demonstrating causation and optimizing physical activity interventions to enhance physical and psychological health for AYA cancer survivors – a population that has been underrepresented in the literature.

## Supplementary information

**Additional file 1.** CONSORT 2010 checklist of information to include when reporting a pilot or feasibility trial.

## Data Availability

The datasets used and/or analysed during the current study are available from the corresponding author on reasonable request.

## Abbreviations

6MWT: 6-min walk test
AYA: Adolescent and young adult
BCTs: Behaviour change techniques
BMI: Body mass index
EXSEM: Exercise and self-esteem model
HRR: Heart rate reserve
MVPA: Moderate-to-vigorous physical activity
PARmed-X: Physical Activity Readiness Medical Examination Form
PSDQ-S: Physical Self Description Questionnaire Short Form
RCT: Randomized controlled trial
RM: Repetition maximum
SD: Standard deviation

## Footnotes

1 Determined based on potential participants’ responses to a series of physical activity readiness screening questions. If a participant’s responses to screening questions indicated cause for concern, they were required to obtain medical clearance from their healthcare provider using the Physical Activity Readiness Medical Examination Form (PARmed-X) prior to participating in the study.

2 The first author is a certified personal trainer who had 6 years experience conducting physical activity interventions with cancer survivors at the time of the study.

3 When systolic blood pressure was ≥ 160 mmHg and/or diastolic blood pressure ≥ 90 mmHg, medical clearance from participants healthcare provider was required using the PARmed-X and the test of aerobic capacity (i.e. 6MWT) was skipped to reduce the risk of myocardial infarction [54].

4 Due to space constraints in their home, two participants completed the aerobic assessment outdoors.
